# Id1 Promotes Tumor Cell Migration in Nonsmall Cell Lung Cancers

**DOI:** 10.1155/2010/856105

**Published:** 2010-04-18

**Authors:** Raka Bhattacharya, Jeanne Kowalski, Allison R. Larson, Malcolm Brock, Rhoda M. Alani

**Affiliations:** ^1^The Sidney Kimmel Comprehensive Cancer Center at Johns Hopkins, Laboratory of Cutaneous Oncology, Johns Hopkins University School of Medicine, Baltimore, MD 21231-1000, USA; ^2^Department of Oncology, Johns Hopkins University School of Medicine, Baltimore, MD 21205, USA; ^3^Department of Surgery, Johns Hopkins University School of Medicine, Baltimore, MD 21287, USA

## Abstract

Id1, which belongs to the Id family of helix-loop-helix transcription factors has been most associated with tumor progression and metastatsis; however, its significance in lung cancers has not been extensively explored. Here we seek to evaluate the expression of Id1 in a pilot study of nonsmall-cell lung cancers (NSCLCs) and determine its diagnostic and functional significance in these tumors. Paired normal and malignant lung tissues as well as a panel of NSCLC primary tumors and cell lines were evaluated for Id1 expression using Western blotting and quantitative RT-PCR. Functional assays were performed to evaluate the role of Id1 in tumor cell growth, migration and progression. We find Id1 expression is upregulated in squamous cell carcinoma when compared to adenocarcinoma of the lung and that expression of Id1 versus the normal control is variable in NSCLCs. We also note that Id1 expression in NSCLC cells is largely growth factor dependant and constitutive expression of Id1 in NSCLC cells significantly increases tumor cell migration without affecting cell proliferation. We conclude that Id1, as a mediator of tumor cell migration, may be an indicator of aggressive potential in nonsmall-cell lung cancers.

## 1. Introduction

Lung cancer is the most common cause of cancer deaths in the world with over one million new cases diagnosed per year [1, 2]. Nonsmall cell lung cancer (NSCLC) accounts for approximately 80% of all lung cancers and is comprised predominantly of adenocarcinomas and squamous cell carcinomas [3]. The major form of curative treatment for NSCLC is surgical resection at an early stage of the disease since systemic therapies for advanced lung cancer show poor objective response rates [4, 5]. Furthermore, evaluating the available biomarkers for NSCLC may predict tumor response to systemic therapy.

Id1 is a member of the helix-loop-helix (HLH) family of transcriptional regulatory proteins which consist of four members, Id1 through Id4 [6]. Of all the Id genes, Id1 has been most closely linked to tumorigenesis since it has been shown to regulate cellular senescence, cellular proliferation, and cell survival [7–10] and has been found to be highly expressed in several human cancers [11–22]. Despite compelling data suggesting a role for Id genes in the development and progression of a large number of human cancers [22], the role of Id genes in lung cancers has not been extensively evaluated to date. A recent study identified Id1 as being differentially expressed in small cell lung cancers [23] and went on to find elevated Id1 gene expression in tumor cells versus matched control tissues; however, no assessments were made for Id1 in nonsmall cell lung cancers.

Here, we seek to define the diagnostic significance of Id1 expression in NSCLCs by exploring the expression patterns for Id1 in primary human tumors and matched normal tissues. We also seek to determine the functional significance of Id1 expression in NSCLC development and progression using in vitro model systems for tumor cell growth and migration using lentiviral-mediated constitutively expressed Id1. We identify a wide range of Id1 expression patterns in NSCLCs without any notable association of expression level with tumor staging or outcome; however, we do note significant effects of Id1 expression on tumor cell migration in vitro. We conclude that Id1 regulates tumor cell migration in NSCLC cells which suggests a functional role in tumor progression for this aggressive form of lung cancer.

## 2. Materials and Methods

### 2.1. Cell Lines and Cell Culture

Nonsmall cell lung cancer cell (NSCLC) lines were obtained from American Type Culture Collection (ATCC, Manassas, VA). The cells H460, A427, H520, H23, H1915, H1299, and U1752 were cultured in RPMI 1640 (Invitrogen, Carlsbad, CA) supplemented with 10% heat inactivated fetal bovine serum (FBS) from Hyclone (Logan, Utah) and 5% Pennicilin-Streptomycin (Invitrogen) at 37°C. *Lentivirus* was produced in HEK 293T cells using previously described protocols [24]. All lentiviral constructs were labeled with GFP and produced in HEK 293T cells. Viral supernatant was collected for 3 days and concentrated using Centricon centrifugation. Viral titer was measured using Flow Cytometry and evaluating GFP expression. Cells were then transduced as mentioned in the manuscript at an MOI of 10. H23 cells were infected by adding viral particles to media along with polybrene at 6 mg/mL and incubating the cells for 6 hours. Fresh media were added and the cells were allowed to recover and grow for a week before they were used for any experiments.

### 2.2. Western Blot Analysis

Cells were washed with phosphate buffered saline (PBS) and pelleted after trypsinization. Proteins were extracted from whole cell lysate by resuspending cell pellets in lysis buffer (250 mM NaCL, 50 mM Tris-Hcl, 5 mM EDTA and 0.1% NP-40) including protease inhibitors obtained commercially (Sigma) and 1 mM PMSF. Protein concentration was measured using the protein assay kit (Bio-Rad, Hercules, CA). 20 ug of protein samples were separated using 15% sodium dodecylsulphate-polyacrylamide gel (SDS-PAGE) commercially available from Bio-Rad for electrophoresis and transferred to Immobilon-P a nylon membrane (Immobilon, Bradford, MA). The membrane was then incubated with primary antibody for 1 hour at room temperature against Id-1(sc-488) and B-actin (sc-1616) (Santa Cruz Biotechnology, Santa Cruz, CA). After washing with TNET (200 mM Tris-Hcl, 50 mM EDTA, 1 M NaCl, 0.5% Tween) the membrane was incubated with secondary antibody against rabbit IgG and the signals were visualized using ECL Western Blot Analysis system (Amersham, Piscataway, NJ).

### 2.3. MTS Proliferation Assay

A colorimetric-based kit One Solution Cell proliferation Assay composed of the novel tetrazolium compound, 3-(4,5-dimethylthiazol-2yl)-5-(3-carboxymethoxyphenyl)-2-(4-sulfophenyl)-2H-tetrazolium, inner salt (MTS) (Promega Corporation, 2800 Woods Hollow Rd, Madison, WI), was used to quantify cell proliferation following the manufacturer's protocol. H23, nonsmall cell lung cancer cells were plated in triplicate with serial dilutions for the standard curve while a fixed number of cells were plated and analyzed for proliferation at various time points.

The MTS reagent was added to cell media and incubated for 1–4 hours and the absorbance measured in a 96-well plate reader at 490 nm. The assay was repeated a minimum of 3 times for each sample.

### 2.4. Scratch/Migration Assays

H23 cells were plated in 12-well plates in duplicate at approximately 85% confluency and allowed to grow overnight. The following day a scratch was made through the center of each well using a 200 *μ*L pipet tip. The cells were washed with PBS and fresh media were added to remove any loose cells. Photographs were taken of the initial scratch and then at regular intervals in order to measure the migration of cells into the scratched area. This assay was performed in triplicate.

A transwell migration assay was performed using Falcon cell culture inserts (Becton Dickinson Labware, Franklin Lakes, NJ). Briefly, 3 × 10^4^ H23 cells were plated in a 24-well cell culture insert in 0.5 mL of 1% FBS-RPMI media. Inserts were then placed in 0.75 mL of 10% FBS-RPMI media in the well and allowed to grow for 48 hours. The inserts with H23 cells were then fixed in 70% ethanol for 3 minutes, rinsed in distilled water, stained in hematoxylene for a minute and then washed again in tap water. Cells within the insert were scraped out with a Q-tip such that only the cells that have migrated through the pores of the insert would remain fixed and stained. Finally, the inserts were cut out and placed on glass slides with fixed by cover slips. Five fields were selected and counted at high power for an average number of cells in each insert.

### 2.5. Human Lung Tissue Samples

Snap-frozen human lung tissues were obtained from the Department of Pathology at Johns Hopkins Hospital under institutionally-approved protocols. A total of 45 tissues that were received in two batches. The first batch of 30 lung tissues contained tumor with adjacent normal (noncancerous) tissue from each patient. The second batch of 15 samples had tumor tissue only from each patient. Annotated data for each tissue specimen include the tumor type, the gender and age of the patient, the smoking status of the patient, and the stage of the disease at the time of diagnosis including the involvement of lymph nodes. 

### 2.6. Quantitative RT-PCR

Total RNA was extracted from the lung tissue samples using the RNEasy mini kit (Qiagen, Turnberry Lane, Valencia, CA). 5 ug RNA was reverse transcribed using SuperScript^TM^ 11 reverse transcriptase with Oligo (dT) (Invitrogen, Carlsbad, CA). Primers for human Id1 included (Fwd: CACCCTCAACGGCGAGAT, Rev: CCACAGAGCACGTAATTCCTC). The sequence for the specific Id1 probe was 56-FAM/ACGGCCGAGGCGGCATGCGT/36-TAM. Quantitative Real-time Polymerase chain Reaction (QRT-PCR) was performed using 10 ng of RNA from each patient sample. TaqMan 2X master mixture (Applied Biosystem) was used in a total volume of 20 uL reaction mixture. Human B-actin was used as the control with commercially available primers and probes (Applied Biosystems). Reaction conditions were 2 minutes at 50°C, 10 minutes at 95°C, 40 cycles of 15 seconds at 95°C, and 45 seconds at 60°C. Every sample was repeated in triplicate and results were confirmed by repeating the entire experiment at least twice.

### 2.7. Statistical Methods

For human lung cancer tissues with paired normal tissue, we tested the first hypothesis of mean differences in Id1 expression levels between cancerous and matched normal tissue by tissue type. Additionally, we examined the effect of patient characteristics, gender (male versus female) smoking status (smoker versus nonsmoker), and the clinical outcome of alive versus dead as of 2001 on mean differences in Id1 expression levels between cancerous and matched normal tissue using a linear model with generalized estimating equations to account for the matching of samples within a patient. For human lung tissues with only cancer tissues (unmatched samples) we tested the hypothesis of mean differences in Id1 expression levels between adenocarcinoma versus squamous cell carcinomas. We used a normal tissue that we purchased from Clonetech and tested the hypothesis that there is a difference in Id1 levels between the cancerous and normal tissues.

## 3. Results

### 3.1. Id1 Expression in Nonsmall Cell Lung Cancer Cells (NSCLC) Is Growth Factor-Dependent

**Figure 1:**
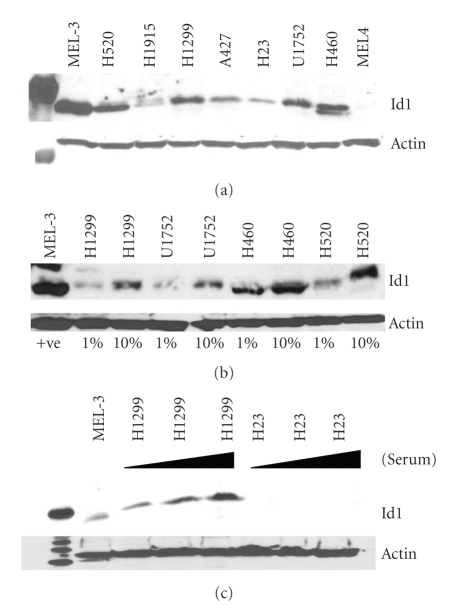
Id1 expression is variable in NSCLC cell lines and responsive to serum induction. (a) Western blot analysis of Id1 expression in nonsmall cell lung cancer (NSCLC) cell lines. Variable expression of Id1 is noted in NSCLCs with elevated expression seen in H520, H1299, U1752, and H460 cells. Mel-3 is used as a positive control for Id1 and Mel-4 is used as a negative control for Id1. (b) Westerm blot analysis of Id1 expression in NSCLC in response to 1% and 10% of serum concentration. (c) Western blot analysis of Id1 expression in H1299 and H23 NSCLC cell lines in response to 1%, 5%, and 10% serum. Mel-3 is used as a positive control for Id1.

In order to explore Id1 protein levels in nonsmall cell lung cancers, a panel of NSCLC cell lines was analyzed for Id1 expression by Western blotting and Id1 transcript level was determined by quantitative RT-PCR. We found variable expression of Id1 in the tumor cell lines evaluated with highest expression in H520, H1299, U1752, and H460 (Figure 1(a)). The lowest level of expression was found in the adenocarcinoma cell line, H23 (Figure 1(a)). Since Id1 gene expression is notorious for being serum-responsive [25–27], we evaluated the effects of varying serum concentrations on NSCLC expression of Id1. We found that the vast majority of NSCLC cell lines evaluated expressed low levels of Id1 in media containing 1% serum and that Id1 was significantly elevated in cells cultured in media with 10% serum suggesting that Id1 expression in NSCLC is growth factor-responsive (Figure 1(b)). Interestingly, while H1299 cells expressed increasing amounts of Id1 as a function of increasing serum concentrations, the H23 adenocarcinoma cell line failed to demonstrate significant Id1 induction following serum exposure (Figure 1(c)). 

### 3.2. Id1 Promotes Migration of Nonsmall Cell Lung Cancer Cells

**Figure 2:**
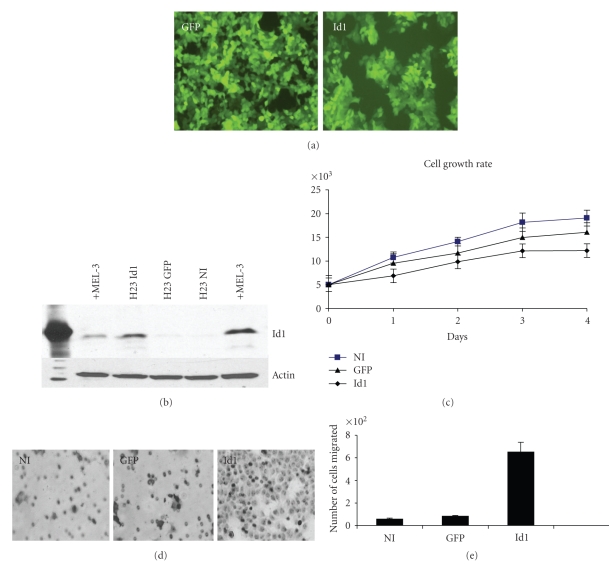
*Exogenous expression of Id1 in NSCLC cells promotes tumor cell migration but not tumor cell growth.* (a) Expression of vector alone (GFP) and Id1 (Id1) in H23 cells infected with a GFP-expressing lentiviral vector. (b) Western blot analysis of Id1 expression in H23 cells following transduction by *lentivirus* expressing Id1 (H23 Id1), GFP alone (H23 GFP), or noninfected cells (H23NI). Mel-3 is used as a positive control for Id1. (c) H23 NSCLC cell growth following transduction with *lentivirus* expressing Id1 (Id1), GFP alone (GFP), and noninfected cells (NI). Error bars indicate standard deviation. (d) Boyden chamber migration assay for H23 NSCLC cells expressing GFP alone, Id1, or noninfected cells. (e) Quantification of Boyden chamber assay depicted in (d).

In order to assess the function of Id1 in NSCLC cells we selected the H23 cell line to ectopically express Id1 and evaluate the biologic consequences. H23 cells were transduced with Id1 using a bicistronic lentiviral expression system also encoding GFP and a GFP lentiviral vector as a control as previously reported [24, 28]. H23 cells were efficiently transduced by the lentiviral vectors as assessed by GFP expression (Figure 2(a)) and Id1 expression was efficiently induced as demonstrated by Western blotting for Id1 (Figure 2(b)). 

In order to evaluate the influence of Id1 expression on NSCLC tumor cell growth, normal H23 cells (NI) and H23 cells transduced with *lentivirus* expressing GFP alone (GFP) or GFP-Id1 (Id1) were evaluated in an MTS proliferation assay. Cells expressing Id1 did not demonstrate any notable change in growth versus GFP-expressing cells, and both transduced cell lines were found to grow more slowly than the noninfected tumor cells (Figure 2(c)). Since Id1 has been implicated in promoting tumor cell migration and invasion [29–31], cellular migration was evaluated in H23 cells expressing Id1 in both a scratch assay and a transwell migration assay. We notably found a significant increase in cellular migration in both a transwell migration assay (Figures 2(d) and 2(e)) and a scratch assay (data not shown) with a greater than 6-fold increase in migration seen in H23-Id1 cells.

### 3.3. Id1 Is Variably Expressed in Nonsmall Cell Lung Cancers

**Figure 3:**
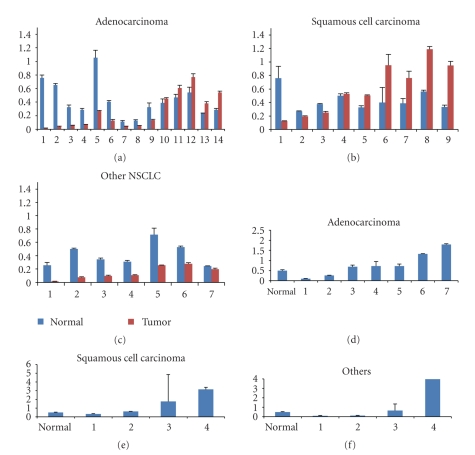
(a) Quantification of Id1 expression by qRT-PCR in primary lung adenocarcinomas versus matched normal tissues. (b) Quantification of Id1 expression by qRT-PCR in primary lung squamous cell carcinomas versus matched normal tissues. (c) Quantification of Id1 expression by qRT-PCR in primary NSCLCs (not adenocarcinoma or squamous cell carcinoma) versus matched normal tissues. (d) Quantification of Id1 expression by qRT-PCR in primary lung adenocarcinomas without matched normal tissues. (e) Quantification of Id1 expression by qRT-PCR in primary lung squamous cell carcinomas without matched normal tissues. (f) Quantification of Id1 expression by qRT-PCR in primary NSCLCs (not adenocarcinoma or squamous cell carcinoma) without matched normal tissues.

In order to evaluate the in vivo expression of Id1 in NSCLC we prepared mRNA from primary human tumors and matched normal tissues and assessed Id1 expression using quantitative RT-PCR. For 30 specimens, each tumor specimen was provided with adjacent normal lung tissue and Id1 expression was compared between tumor and normal tissue from each patient (Figures 3(a)–3(c)). Since the vast majority of specimens were lung adenocarcinomas (AD) or squamous cell carcinomas (SCC), tumors were sorted based on this distinction to further evaluate Id1 expression trends. Among the 14 lung adenocarcinomas, we observed elevated Id1 expression in tumor specimens versus normal lung in 5 cases with no notable association with tumor stage at the time of diagnosis (Figure 3(a)). We also noted overall significantly lower Id1 expression as compared to matched normal tissue (*P* = .02) in adenocarcinomas (Table 1). Similarly, among the 9 matched squamous cell carcinomas evaluated elevated, Id1 expression was found to be elevated in tumor specimens versus normal lung in 5 cases with no notable association with tumor stage at the time of diagnosis (Figure 3(b)). In contrast, for the 9 matched squamous cell carcinomas of the lung, we observed overall greater average Id1 expression as compared to matched normal tissue, although these differences were not statistically significant (*P* = .65). Furthermore, among the 7 other matched lung cancer tissue types (2 carcinoid, 1 carcinoma, 1 small cell, 1 small and large cell, 1 nonsmall cell, and 1 bronchoalveolar) mean Id1 expression was significantly lower in tumor as compared to matched normal tissue (*P* = .007) (Figure 3(c)). 

Comparison of Id1 expression in unmatched lung adenocarcinomas and squamous cell carcinomas showed no statistically significant differences in mean Id1 expression (Figures 3(d)–3(e), Table 2), with variable expression in tumor specimens relative to a normal lung control. For the 4 unmatched “other” NSCLC tumors, only 1 tumor demonstrated significantly elevated Id1 expression with 20-fold increased Id1 expression versus the normal control noted in a carcinoid tumor (Figure 3(f)).

**Figure 4:**
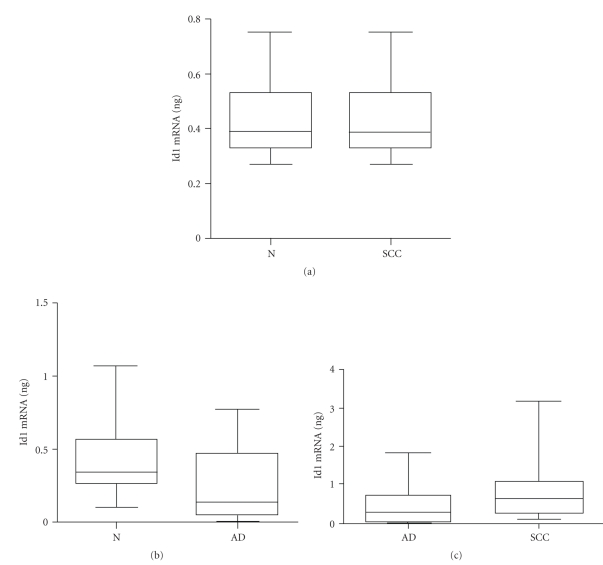
Boxplots comparing Id1 expression levels for (a) squamous cell carcinomas of the lung versus matched control tissue, (b) adenocarcinomas of the lung versus matched control tissue, and (c) squamous cell carcinomas of the lung versus adenocarcinomas of the lung.

 Boxplot analyses for all specimens did not demonstrate significant differences between either lung adenocarcinomas or squamous cell carcinomas and normal tissues (Figures 4(a) and 4(b)); however, statistically increased Id1 expression was nearly achieved for squamous cell carcinomas versus adenocarcinomas (Figure 4(c)) (*P* = .0579) (Tables 1 and 2).

 Because of the greater number of samples available from the matched tissues, we further examined the effect of other patient characteristics, gender (male versus female), smoking status (smoker versus nonsmoker), and patient outcome (alive versus dead), on mean differences in Id1 expression levels with respect to each tissue type, adenocarcinoma, squamous cell carcinoma, and other (Table 3). No statistically significant mean Id1 expression differences were observed for each comparison, although increased Id1 expression in adenocarcinoma patients who reported smoking was nearly significant (*P* = .06) as compared to reported nonsmoker adenocarcinoma patients.

## 4. Discussion

Id proteins have been shown to be highly expressed in a large number of cancers (reviewed in [22]). In several of these cancer types, overexpression of Id1 is associated with an aggressive phenotype and poor clinical outcome [14–16, 32]. Functional studies have demonstrated that Id1 is able to promote cell proliferation [33], delay senescence in primary human cells [7, 9], and promote cell migration [34] and the metastatic phenotype of cancers [35, 36]. While Id proteins, and Id1 in particular, have been found to be upregulated in a large number of tumors, studies of Id genes in lung cancer have been limited to date with a single previous study evaluating Id gene expression in small cell lung cancers focused on Id genes as biomarkers in these tumors rather than the functional effects of Id genes in this malignancy [23]. Here we have evaluated the expression of the helix-loop-helix transcription factor, Id1, in human nonsmall cell lung cancers and its functional significance in these tumors. Our study indicates that although significant Id1 expression is seen in NSCLCs, no particular trends are noted in comparison to matched normal human tissues. Interestingly, our study indicates that patients with lung adenocarcinomas express lower levels of Id1 when compared to patients with squamous cell carcinomas although neither level is consistently above that expressed in surrounding normal tissues. 

Our in vitro analysis of Id1 function in NSCLC cell lines indicates that although most tumor cells expressed a high level of Id1 when cultured in media containing 10% serum, most Id1 expression in these tumors is growth factor dependent and not constitutive. Of note, H23 adenocarcinoma cells were shown to express low levels of Id1 even when grown in media containing increased levels of serum. We took advantage of this attribute of this particular cell line to ectopically express Id1 and evaluate its functional role in NSCLC. Our study demonstrated that although the growth rate of cells expressing ectopically transduced Id1 was not significantly altered by Id1 expression, Id1-transduced cells demonstrated a significantly increased rate of migration when compared to cells that were noninfected or expressed only GFP.

Studies of Id1 expression in primary human NSCLCs demonstrated lower Id1 expression in adenocarcinomas of the lung when compared to squamous cell carcinomas with variable expression compared to matched normal lung tissues. 

Given the above data, we hypothesize that deregulated expression of Id1 in NSCLCs may be associated with an increased rate of cell migration and therefore contribute to metastasis in patients with poor outcome. While the prognosis for the vast majority of NSCLC patients is extremely poor, we note that the primary human tumors evaluated in this study segregated into groups with elevated tumor Id1 expression versus normal tissue and decreased tumor expression versus normal tissue. We therefore suggest that Id1 expression in NSCLCs may allow for distinction of NSCLC tumor subtypes which may be specifically responsive to particular forms of therapy targeting Id1. Given the universally poor prognosis of NSCLCs and the lack of an effective therapy for advanced disease, the results of this pilot study should pave the way for larger-scale evaluation of Id1 in NSCLCs to assess its true significance in this malignancy.

## Figures and Tables

**Table 1:** Id1 expression level in primary human lung tissues versus adjacent normal tissue. Expression level is average qRT-PCR signal for Id1/average signal for GAPDH × 10^3^.

Tissue type	No. samples	Mean (SE)* Id1 expression		95% C.I	*P*-value^+^
		Normal	Tumor		
Adenocarcinoma	14	2.55 (0.07)	2.14 (0.14)	(0.09, 0.74)	.02
Squamous Cell Carcinoma	9	2.62 (0.05)	2.68 (0.12)	(−0.36, 0.24)	.65
Others	7	2.58 (0.07)	2.03 (0.16)	(0.21, 0.90)	.007

*Natural log transformed mean and standard error (SE); ^+^ *P*-value based on two-sided two-sample *t*-test of hypothesis of no mean difference between tissue types.

**Table 2:** Id1 expression level in primary human lung adenocarcinomas versus squamous cell carcinomas. Expression level is average qRT-PCR signal for Id1/average signal for GAPDH × 10^3^ Primary NSCLC tumor specimens were evaluated for Id1 expression by qRT-PCR and expression levels for SCCs and AdenoCAs were compared.

Tissue type	Mean (SE)* Id1 expression	Tissue type	Mean (SE)* Id1 expression	*P*-value^+^
Adenocarcinoma	2.76 (0.16)	Squamous Cell Carcinoma	3.01 (0.22)	.41

*Natural log transformed mean and standard error (SE); ^+^ *P*-value based on two-sided two-sample *t*-test of hypothesis of no mean difference between tissue types.

**Table 3:** Covariate analysis of Id1 expression in NSCLCs and its association with gender, smoking and patient clinical status.

	Gender			Smoke			Alive		
	Male (s.e)	Female (SE)	*P*-value	Nosmoke	Smoke	*P*-value	Death	Alive	*P*-value
Adeno	2.48 (0.15)	2.30 (0.09)	.32	2.14 (0.00)	2.32(0.10)	.06	2.44 (0.11)	2.31 (0.16)	.38
Squamous	2.68 (0.08)	2.60 (0.08)	.45	2.74 (0.00)	2.66(0.06)	.21	2.63 (0.07)	2.66 (0.10)	.80
Others	2.39 (0.08)	2.24 (0.15)	.36	2.44 (0.30)	2.25(0.11)	.27	2.24 (0.19)	2.35 (0.08)	.58
